# Understanding Reciprocity Among University Students in Low-Resource Settings: Validation and Measurement Using a Mixed-Methods Approach

**DOI:** 10.3389/fpubh.2022.922892

**Published:** 2022-06-03

**Authors:** Mahmoud M. AbuAlSamen, Tamam El-Elimat

**Affiliations:** Faculty of Pharmacy, Jordan University of Science and Technology, Irbid, Jordan

**Keywords:** effort-reward imbalance, reciprocity, mixed-methods research, factor analysis, academic-related stress

## Abstract

**Objectives:**

This study aimed to investigate reciprocity among university students in low-resource settings using a convergent mixed-methods approach in Jordan. The study operationalized the effort-reward imbalance (ERI) model which is a sociological framework used to predict occupational-related health outcomes. The basic theory of ERI model assumes that an imbalance of effort and reward predicts adverse health outcomes.

**Methods:**

The research involved two studies, Study I (*n* = 833) to quantitatively measure ERI and Study II to collect qualitative data (*n* = 44) on the drivers of ERI among university students. In Study I, a modified Arabic version of the ERI questionnaire was used. The study measured ERI and investigated the reliability and validity of the Arabic version of the ERI model questionnaire. In Study II, data were collected from focus groups and personal interviews and thematic analysis was used.

**Results:**

The results suggested that ERI was associated with poor academic performance (OR=2.31, 95% CI 1.60–3.32), absenteeism (OR=1.66, 95% CI 1.21–2.27), low exercise level (OR=2.02, 95% CI 1.49–2.74) and poor self-reported health (OR=1.12, 95% CI 1.08–1.30). Three major themes emerged, namely high academic load, financial pressures and negative influence on the students' performance, wellbeing and health to explain effort-reward imbalance.

**Conclusions:**

Results suggest that ERI among university students is multi-faceted and is not bound only to academic-related demands and that the extrinsic factors such as the economic context of Jordan is among drivers of ERI.

## Introduction

Reciprocity is a notion that determines the balance between efforts and rewards. The effort-reward imbalance model (ERI) was proposed by Siegrist et al. to predict occupational-related health outcomes (1). Whilst reciprocity assumes that ‘high' effort should adequately be compensated with ‘high' reward, negative emotions and stress may be elicited when this equilibrium is violated (i.e. *high* effort with *low* reward) (2). The ERI model emphasizes the significance of the social role of the subject (3). When people exert their efforts as per their social roles, the social role is expected to satisfy their self-regulatory needs such as successful performance, recognition, integration, and the well-being (1–4).

Following Siegrist's seminal work, the ERI model has been replicated in many different populations worldwide (5). The model was mainly used to investigate the effect of work conditions on the subject and was demonstrated to be ubiquitous to researchers as it was successfully validated in more than ten languages (6, 7). Much of the research conducted on the ERI model was mainly produced from the USA, Europe, and Eastern Asia (2). However, little work has been done on exploring the validity of the ERI model in the Middle East (8). While much of ERI research was investigated in workplace conditions, there has been a growing interest in understanding the validity of ERI model when applied to other social settings such as schools and universities. There has been no work done on understanding the interaction between effort and reward in university settings among students in Jordan. Moreover, a closer look at the literature on ERI shows that most of the evidence takes the form of quantitative research with very little qualitative research to understand the drivers of ERI.

In this study, we aimed to investigate the validity of the ERI model among university students in Jordan using a mixed-methods design to generate both quantitative and qualitative data. We also explored the potential influences of ERI on students' academic performance, wellbeing and health.

## Materials and Methods

### Design and Study Population

This study was designed to take a convergent mixed-methods approach. The research involved two studies, Study I used cross-sectional questionnaires to collect quantitative data on ERI among university students, and Study II to collect qualitative data on the drivers of ERI. The major aim of using a mixed-methods design is to triangulate the data from the quantitative questionnaire with qualitative findings that can offer depth in interpretation.

All participants of this study were enrolled at Jordan University of Science and Technology (JUST), Irbid, Jordan. In an attempt to make the study participatory, students were involved at different stages of this research including co-designing, co-analysis and interpretation of study results. For instance, students were involved in the analysis of qualitative data from an early stage in the project. After concluding the qualitative study, the results were discussed with other students in a brainstorming session.

The recruitment location was based on student density and diversity to enhance the quality of the sample obtained. JUST is considered to be the most culture-diverse public institution of higher education in Jordan and attracts students from all Jordanian governorates in addition to having students from 60 different international nationalities.

To be eligible for participation in this study, participants should meet the criteria of being (1) enrolled in a full-time undergraduate study program at JUST; (2) at least 18 years or older; (3) be willing to participate in the study.

## Study I: Quantitative Study

Data collection was conducted by dividing the university into several clusters based on schools of study with a total of 11 clusters. We aimed to reduce bias in data collection by sampling students directly from lecture halls of courses which had a policy of compulsory attendance. These courses involved students from different student levels and ensured the highest representation of the student population at JUST. A total of five research assistants approached the student halls, by giving a general exposition of the study aims to each lecture hall. Students who showed interest to participate were approached using paper-based questionnaires. Participating students gave their written consent. No university official or course instructor were involved in the recruitment of students, and all students had the free will to participate or to withdraw their consent.

We estimated the sample size using a precision level of 0.05 and 95% confidence level, to be 379. To account for the clustered design of the study, the estimated sample size was multiplied by design effect (DE) which can be computed using the formula DE = 1+ (*m*- 1) × *ICC*, where *m* is the number of participants sampled per each school and ICC denoting the intra-cluster correlation coefficient. The intra-cluster correlation coefficient determines the variance within clusters in relation to the variance between clusters and in the current study it was estimated to be 0.02 based on previous research conducted by authors on student samples at JUST. We aimed to sample 50 students from each school. Hence, sample size was determined to be at least 750. We invited 1000 students to participate, and between May and July 2018, a total of 833 participants completed the questionnaire. Non-response was often due to lack of time by students to participate.

### Instrumentation

The development of the Arabic ERI questionnaire in university students was based on the existing body of literature and published guidelines on the ERI model (2, 9).

The survey was developed in Arabic using available translation of the 2013 Arabic version and previous study done on students in school settings (8, 10). In the first stage, the questionnaire was piloted on 89 students for clarity of wording and expression. The pilot sample was not involved in any further analysis. There were four items in the effort scale (E1–E4), 10 items in the reward scale (R1–R10), and five items in the overcommitment scale (OC1–OC5). Newer guidelines recommended the use of condensed response scales (four-point Likert scale instead of 5-point) for obtaining higher response rate (9). The three dimensions were surveyed using a four-point anchored scale (strongly agree = 4, agree, disagree, and strongly disagree = 1).

### Measures

The following measures were obtained in this study: effort-reward imbalance (ERI) ratio, academic performance, absenteeism, exercise level and self-reported health.

### Effort-Reward Imbalance

The effort reward imbalance is computed by dividing the total effort scale over the reward scale. This ratio is then corrected with correction factor *k*, that is found by dividing the number of reward items by the number of effort items. Correction factor *k* in this study was 2.5 (9).

### Academic Performance

Classification of academic performance was based on JUST internal grading system. Participants were asked to provide data on their GPA and a cut-off point of 2.75 GPA out of 4.00 was taken, where <2.75 was considered a poor academic performance and above was considered good academic performance.

### Absenteeism

Students at JUST attend classes on campus from Sunday to Thursday. Participants were asked to indicate how many days they had to attend university in their current semester of study, in addition to how many days they usually missed on a weekly basis. Absenteeism was computed as the percentage of how many days students were absent on weekly basis out of the total days of compulsory attendance. Participants were then classified into either showing absenteeism (at least absent a day every week) or not showing absenteeism.

### Exercise Level

Participants were asked to indicate how many times per week they were engaged in exercise activity. Low exercise level was defined as having <150 min per week of exercise, while those doing more than 150 min were defined with good level of exercise.

### Self-Reported Health

Participants were asked to rate their health on a scale from 1 to 7. The scale was classified into three classes: poor health (1–3), fair health (4), good health (5–7).

### Statistical Analysis

Reliability of the effort, reward, and overcommitment scales were evaluated using Cronbach's alpha and McDonalds Omega measures. Factorial structure of the questionnaire was conducted using exploratory factor analysis. To determine the number of factors to be extracted, we employed the Horn's parallel analysis method by running a Monte-Carlo simulation with a randomly generated set of data. Additionally, the number of factors was determined by Kaiser's rule and by inspection of the scree plot at the breaking point. The Kaiser-Meyer-Oklin (KMO) sample adequacy index was set at >0.6. The items were rotated by direct oblimin. The model fit was assessed by chi-squared test, the root means square error of approximation (RMSEA) and Tucker-Lewis index (TLI).

Binary logistic regression models were constructed to investigate the associations between ERI and academic performance, physical activity and absenteeism. Ordinal logistic regression was used to investigate the association of ERI with self-reported health. Questionnaires with data missing on either effort or reward were not considered in the analysis. Statistical significance was set at *p* < 0.05. All data analyses were conducted in R Studio version 1.1.463.

## Study II: Qualitative Study

Data was collected through conducting both personal interviews and focus groups and they were purposefully targeted at students in different years of study (years 1–6). As most students are not obligated to follow university courses plan and may interchange courses between each 2 years, focus groups were conducted with students of each 2 years together (years 1–2), (years 3–4) and (years 5–6). A total of three focus groups and 11 interviews were conducted during April-May 2018.

At the beginning of a focus group or an interview, a research assistant introduced the aims of the study and initiated discussions regarding each student experience and reflections. The open-ended questions that were used to elicit insights from students are described in Table 1.

**Table 1:** Research questions guide in the qualitative study.

Describe the efforts that you make during your university studies in terms of preparation for assessment exams and assignments
Do you feel that the amount of effort you put has changed over the course of your study?
What motivates you to make an effort in your studies?
What does a university degree mean to you and the people close to you?
How do you describe the impact of your family on the effort you make in your studies?
What could change in the effort you make in your studies, if your tuition was covered by your family or by a scholarship?
What do you think is the best reward you deserve for the effort that you put in your studies?
Do you feel that this described reward was realized?
Do you feel rewarded in terms of grades and recognition?
Do you feel recognized by your family and faculty?


Interviews were recorded and verbatim transcribed for coding. Thematic analysis was completed by the lead author in this study with the help of two other research assistant students who conducted the interviews. Coding was done manually without using qualitative data analytic software due to the limited availability of funds. Before coding, researchers familiarized themselves with the interviews and then started identifying themes that were included in the codebook. This codebook has an index of all identified themes that were constantly compared together for refinement. Each transcript was coded, and codes were collectively grouped into major themes and sub-themes.

### Quality Assurance

Quality assurance of the qualitative study on the aspects of credibility, dependability, confirmability, transferability and reflexivity is described in full in Table 2.

**Table 2:** Qualitative study quality assurance measures.

**Criterion**	**Strategy**	**Application**
Credibility	Prolonged engagement and persistent observation	Sufficient time was spent by lead researchers and field researchers in collecting data, gaining deeper insights and develop understanding and context of the data.
	Triangulation	More than one method of data collection was followed in both qualitative and quantitative methods. Data came from more than one source (students in years 1–6) and conducted via different instruments (interviews and focus groups).
	Peer debriefing	All steps in this research were supervised by the lead researchers.
	Negative case analysis	Cases which did not conform with our understanding were sought to refine interpretation of data.
Dependability and confirmability	Full description of study design	All research methods are described in sufficient detail in the method section.
	Peer review	All steps of this research including design, piloting, data collection and analysis were reviewed and audited by external experts.
Transferability	Thick description	Investigators described in sufficient detail the context and the full methods followed in this research.
Reflexivity	Diary	The lead researchers recorded their conceptual lens, positionality and any implicit assumptions that might affect data analysis and/or interpretation.

#### Reflexivity Statement

This research was conducted when the lead author was a student at JUST while the second author was his research mentor. Several efforts were made to acknowledge, understand and reduce the possible impact of this studentship on the research and interpretation of data. Much of the qualitative data was analyzed with the help of other students under the guidance of research method experts. The reason of including other students was to make this research a participatory project. However, this may have influenced how data was being analyzed and interpreted. Therefore, after the themes were extracted, two external experts assessed the quality of data analysis and its interpretation. Accordingly, the analysis was refined several times until a common consensus was reached.

#### Ethical Consideration

The study protocol was approved by King Abdallah University Hospital Institutional Review Board at Jordan University of Science and Technology (Approval number: 15/114/2018).

Participants had to sign a written informed consent upon participation.

## Results

### Participant Characteristics

Demographic details of participants are shown in Table 3. Regarding the quantitative arm of the study, the majority of students were females (61.8%) compared to males making up 38.2% of the sample. Around 61.0% of the students were enrolled in undergraduate programs at medical schools from all different study years. There were very few students who were employed at the time of the study. Interestingly, 58.0% of students were enrolled on competitive basis compared to 38.3% of students who pay considerably higher fees for admission within the parallel program. Almost half of the students were self-funded, while around 49.1% were covered by a scholarship. Higher education scholarships in Jordan are usually paid by the Royal Court for the family members of public sector employees, including teachers, in addition to scholarships offered by the military for family members of the Jordanian Armed Forces staff. Nevertheless, family income is variable among students, reflecting their different socioeconomic backgrounds. Similar demographics were shown among the participants of the qualitative arm of the study.

**Table 3:** Demographic characteristics of participants.

**Variable**	**Study I: Quantitative** **(*n* = 833)**	**Study II: Qualitative** **(*n* = 44)**
**Gender, (** ***N*** **%)**		
Male	318 (38.2)	19 (43.2)
Female	515 (61.8)	25 (56.8)
**Age, mean (SD) years/ range**	19.8 (1.5)	18–24
**Field of study**, ***N*** **(%)**		
Sciences, health and medical studies	508 (61.0)	24 (54.5)
Humanities, arts and engineering	325 (39.0)	20 (45.5)
**Academic Year**, ***N*** **(%)**		
First year	285 (34.2)	11 (25.0)
Second year	167 (20.0)	14 (31.8)
Third year	181 (21.7)	9 (20.5)
Fourth year and above	200 (24.0)	10 (22.7)
**Job status**, ***N*** **(%)**		
Full-time job	6 (0.7)	0 (0.0)
Part-time job	52 (6.2)	3 (6.8)
Unemployed	775 (93.0)	41 (93.2)
**Study program**, ***N*** **(%)**		
Competitive	483 (58.0)	25 (56.8)
Parallel	319 (38.3)	14 (31.8)
International	31 (3.7)	5 (11.4)
**Financial aid**		
Self-funded	417 (50.1)	29 (65.9)
Financial aid from military or	409 (49.1)	10 (22.7)
Royal Court		
Foreign funding (international students)	7 (0.8)	5 (11.4)
**Family income**, ***N*** **(%)**		
<400 JD	244 (29.3)	19 (43.2)
400–700 JD	251 (30.1)	13 (29.5)
700–1000 JD	171 (20.5)	9 (20.5)
>1000 JD	167 (20.0)	3 (6.8)
**Residency**		
Northern Jordan	526 (63.1)	30 (68.2)
Central and Southern Jordan	307 (36.9)	14 (31.8)

## Study I: Quantitative Study

### Factorial Structure and Construct Validity of the Arabic ERI Questionnaire in University Students

**Figure 1:**
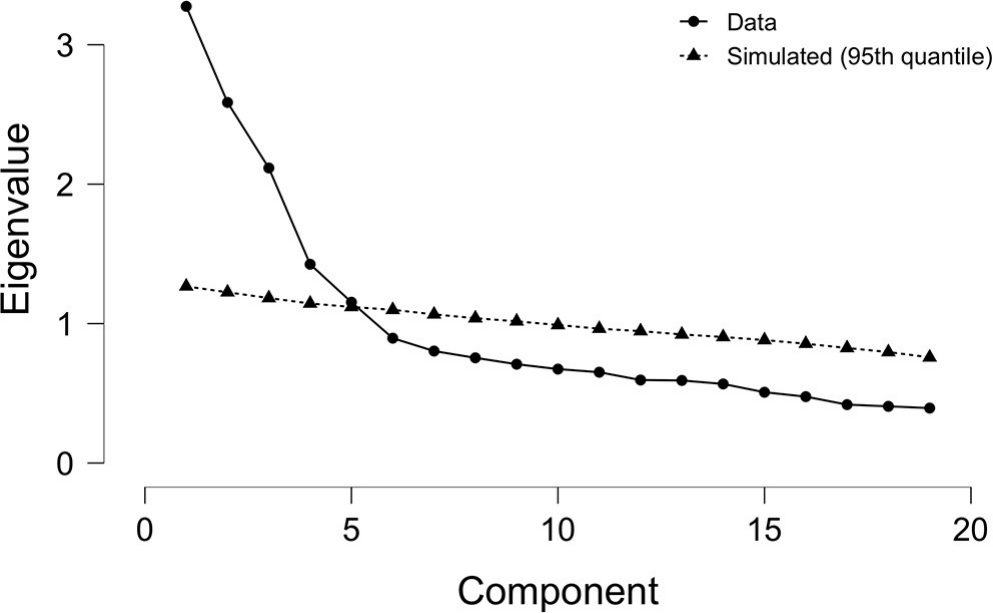
A scree plot from Horn's parallel analysis method supporting a five-factor solution.

All items in effort, reward, and overcommitment scales were entered into exploratory factor analysis. The Kaiser-Meyer-Olkin measure of sampling adequacy was found to be 0.773, which exceeds the 0.6 minimum threshold. The Bartlett's test of sphericity was significant at *X*^2^ (171), *p* < 0.0001, which renders the scales eligible for factorability. All communalities were higher than 0.60. At first, Horn's parallel analysis method was used to identify the number of factors to be extracted. Results from this analysis suggested a five-factor solution, as shown in Figure 1. This solution showed that both effort and overcommitment scales were unidimensional, while reward scale loaded on three separate factors. Furthermore, we determined the number of factors to be extracted based on Kaiser's rule of extracting factors with eigenvalues greater than 1. A three-factor solution was supported with three unidimensional scales. Similar results were obtained by inspecting the scree plot at the breaking point. For the purpose of further exploration of possible solutions, we also forced a four-solution on the data. We investigated the fit indices of the three models as shown below. The results suggested that the fit indices were substantially improved in a five-factor solution compared to three and four factor solutions (Table 4).

**Table 4:** Model fit indices for the proposed three, four and five-factor solutions.

**Solution**	***X*** **^2^**	***df***	**RMSEA (95% CI)**	**TLI**
3-Factor	766.65	117	0.082 (0.076–0.087)	0.706
4-Factor	360.75	101	0.056 (0.049–0.062)	0.864
5-Factor	185.82	86	0.038 (0.03–0.045)	0.938

As the five-factor model showed the best fit to the data, we opted for this solution. The rotated structure matrix is shown in Table 5, along with reliability measures for each of the three scales. On the factors 1 and *2*, the items for effort (E1, E2, E3, and E4) and overcommitment (OC1, OC2, OC3, OC4, and OC5) loaded respectively. Items measuring reward loaded on three different factors. On *factor 3*, items (R7, R8, and R9) loaded strongly and positively and hence this factor was named as ‘*security'*. On *factor 4*, items (R2, R5, R6, and R10) loaded positively and this factor was named as ‘*academic esteem'*. On *factor 5*, items (R1, R3, and R4) loaded strongly and positively, and therefore this factor was named as ‘*academic support'*. From the data shown, the Arabic ERI questionnaire in university students fits a five-solutions model explaining 56% of the variance (Table 5).

**Table 5:** Reliability measures and factorial structure of the effort, reward and overcommitment scales.

**Items**		**Cronbach alpha**	**McDonald's omega**	**Factorial loading**				
				**F1**	**F2**	**F3**	**F4**	**F5**
**Effort**		0.73	0.81					
E1	I have constant time pressure due to a heavy study load			0.70	–	–	–	–
E2	My study load has become more and more demanding			0.72	–	–	–	–
E3	I am under constant pressure to pass with highest grades			0.81	–	–	–	–
E4	I am under constant pressure in my studies to secure a job			0.68	–	–	–	–
**Reward**		0.76	0.87					
R1	I receive the respect I deserve from my instructors			–	–	–	–	0.72
R2	I receive the respect I deserve from my parents			–	–	–	0.63	–
R3	I receive proper support in difficult tasks or courses			–	–	–	–	0.70
R4	When I need help, I can get it from my instructors			–	–	–	–	0.73
R5	Overall, my level is good in all courses			–	–	–	0.70	–
R6	I feel that my specialty at the current institution reflect my hard work in the past and at the present			–	–	–	0.43	–
R7	I feel unfit to my current field of study			–	–	0.70	–	–
R8	I feel that studying is useless			–	–	0.83	–	–
R9	I do not think that my university studies should occupy my first priorities			–	–	0.78	–	–
R10	I feel satisfied about my academic performance			–	–	–	0.72	–
**Overcommitment**		0.69	0.80					
OC1	As soon as I get up in the morning, I start thinking about study problems			–	0.57	–	–	–
OC2	Studying rarely leaves my mind; it is still on my mind when I go to bed			–	0.79	–	–	–
OC3	People close to me say I sacrifice too much for my study			–	0.55	–	–	–
OC4	I miss some lectures regularly			–	0.69	–	–	–
OC5	If I postpone something that I was supposed to be done today, I will have trouble sleeping at night			–	0.69	–	–	–
			**VE (%)**	18.5	13.7	9.3	8.3	6.1
			**CVE (%)**	18.5	32.2	41.5	49.8	55.9

*F1–F5, factors 1–5 from factor analysis, VE, variance explained; CVE, cumulative variance explained*.

### Reliability of ERI Arabic Questionnaire

The reliability measures for all items in the effort, reward, and overcommitment scales are given in Table 5. The results suggested that the three scales have satisfactory reliability and internal consistency for measuring effort-reward imbalance in university students in Jordan.

### ERI Ratio

The mean corrected effort-reward imbalance was 1.19 with a standard deviation of 0.43. This ratio can be interpreted as for every 1.19 units of effort students make, a 1 unit of reward was reciprocated.

### Association of ERI With Academic-Related Performance and Exercise

The associations between ERI and academic performance, absenteeism, low physical activity, and self-reported health were explored. The results are shown in Table 6. The odds of poor academic performance were 2.31 higher in students showing an imbalance between effort and reward, compared to those who did not. Similarly, students with ERI showed higher odds of absenteeism and also low exercise level. Moreover, ERI was associated with poor self-reported health.

**Table 6:** Unadjusted and adjusted odds ratios of the association of effort-reward imbalance with poor academic performance, absenteeism, and self-reported health.

**Outcome**	**Unadjusted OR (95% CI)**	***p*** **-value**	**Adjusted OR (95% CI)**	***p*** **-value**
Poor academic performance	2.19 (1.55–3.08)	<0.0001	2.31 (1.60–3.32)	<0.0001
Absenteeism	1.60 (1.18–2.17)	0.003	1.66 (1.21–2.27)	0.002
Low exercise level	2.05 (1.51–2.77)	<0.0001	2.02 (1.49–2.74)	<0.0001
Poor self-reported health	1.20 (1.01–1.45)	0.02	1.12 (1.08–1.30)	0.01

*Adjusted for age, gender, specialty, and family income.

## Study II: Qualitative Study

Analysis of the qualitative study data generated the themes and sub-themes shown in Table 7. The first theme relates to the high academic load on students. Most students agreed that university demands make it impossible to maintain a satisfactory performance level without high efforts. This was a common ground for all students, even when they were performing at different academic levels.

**Table 7:** Themes and sub-themes from qualitative study.

**Theme**	**Sub-themes**
Academic demands	Highly demanding academic load of exams and assignments
Financial pressure	Pressure from family as source of funding
	Pressure to maintain scholarship status from granting agencies
	Stress about finding a job post-graduation
	Stress about economic challenges in Jordan
Influence on student	Low reward and reciprocity
	Influence on students' academic performance, health and well-being

Another theme emerged to associate field of study with high efforts. Students recognized that being enrolled in highly respected fields earns them better social acceptability and social capital, and hence, to maintain this social capital, they had to put in more effort. For instance, several students indicated that their families support them because they secured admission in highly respected fields such as engineering or medical studies at a prestigious university in Jordan. If they were not enrolled in these majors, they would have received less support or were not given the opportunity to pursue their education. A student said: ‘*my family respects me because I study in the medical school'*. It should be also mentioned that several students showed how maintaining a studentship in a prestigious university in Jordan in a respected field is of utmost importance to them. This added more evidence to another interconnected theme which demonstrated the role of funding source. It was evident, among students who were not funded by their families, that they were under the stress of maintaining scholarship funding by granting agencies (such as Ministry of Higher Education and Scientific Research, Jordanian Armed Forces, Royal Hashemite Court and local charities). Many students citing financial reasons expressed how difficult it will be for them to pursue their studies if funding was not available. These results suggest that financial challenges are among the most important drivers of high effort and thereby high ERI.

Another sub-theme showed that the context of Jordan influences how students perceived their studies, as some put more efforts to have higher chances of securing jobs after obtaining their degrees. Some students expressed how important it is for them to secure good jobs to support their families. Much emphasis was given on the economic challenges in Jordan and how students viewed their degrees as a ticket to leave Jordan to find jobs abroad. It was evident from the qualitative findings that poor career prospects and the ambiguity about future all result in low levels of reward and security.

A student said in a gendered tone: ‘*my family is supporting me now, so I can support them [later]. As a female, my father accepted that I pursue my studies so I can contribute to the financial needs of our household later'*. Another student said: ‘*there are no available jobs once you graduate. If you want to work abroad, [they] look into your high grades'*, while another added: ‘*all my family members work currently in the Gulf countries and [I] am planning to travel abroad once I finish my studies'*. This was not only limited to the Middle Eastern region, but also encompassed the wider globe: ‘*to secure a scholarship to study a master's degree in Europe or [North] America, you need to be an outstanding student. [My] only way to immigrate is to study abroad'*.

These high efforts were perceived to have low reward in return. For instance, students expressed their frustration over challenging level of assessments and their inability to maintain high academic performance.

A student said: ‘*Whatever I do, I will always get the same grade. Whenever I study and dedicate much of time to my exams, I can hardly pass*'. Moreover, several inputs were made at the influence of high efforts on health and well-being of students. It was highlighted how demanding it is to attend classes daily and how can this push students to skip classes to have more time for exam preparation. The problem of absenteeism was reported by all study participants.

## Discussion

Comparing the quantitative and qualitative results of this study sheds light on the significance of mixed-methods research to help in the interpretation of quantitative data. In this study, we quantified ERI in a sample of university students while offering an account, from the students' perspective, on the drivers of ERI. The qualitative arm of the research has established a link on the intersectionality of socioeconomic inequalities and ERI and the need to examine ERI among university students while considering Jordan as an economically challenged country (11). The research draws our attention to the motivations of high effort which included scholarship status, family support, social capital and not only high demands by academic settings. It also expanded our lens on how students regarded recognition as important as much as grades in an academic context. This study was mostly participatory which meant that its interpretation involved all relevant parties including students, educators and university officials.

The results suggest that the lack of reciprocity may be associated with poor students' health. The germinating corpus of evidence is linking ERI to several diseases and had been used to explain the ‘social gradient' of incidence of these diseases such as heart disease and diabetes II (5, 11–14). Other studies have also highlighted a role of failed reciprocity in developing burnout and serious psychiatric disorders such as depressive and anxiety-like symptoms (15, 16). Our results are congruent with evidence from the literature of both working and non-working contexts. A research study done on school children in China (*n* = 1004) showed that ERI was associated with poor self-reported health (10). Similarly, a study done on Swedish students (*n* = 403) gave evidence of the association between ERI and poor self-reported health and somatic pain (17). In terms of university students, a study conducted in Germany (*n* = 698) correlated ERI to symptoms of poor self-reported health and symptoms of anxiety and depression (18). Results from working contexts showed that ERI was associated with absenteeism when applied to employees (19). Interestingly enough, the link between ERI and poor academic performance and low level of exercise reported in this study were not reported elsewhere, which adds new data to the body of knowledge on the criterion validity of the ERI model.

In terms of the psychometric properties of the Arabic ERI questionnaire among university students, the questionnaire had demonstrated satisfactorily reliability. Recent reports on using ERI in measuring academic-related stress in student settings showed similar and comparable results to the findings of this study (20). The main addition this study adds to the body of knowledge on student ERI is its use of mixed-methods design to supplement the quantitative findings with qualitative data that can offer a depth in interpretation. Moreover, results from Horn's parallel analysis supported a five-factor solution, in which both the effort and overcommitment scales were unidimensional while the reward scale showed a three-factorial structure. The unidimensionality of both effort and overcommitment scales had been well documented in the literature within work settings (2, 9, 21). Worth mentioning that while the chi-squared test was used as a test of model fitness in this study and in earlier research, it had been well established that obtaining a non-significant chi-squared test in relatively larger samples is very difficult (7). In this regard, other model fitness indices reported in this paper added further evidence to the five-factor solution which had documented previously in the literature in working contexts (22–24).

The findings of this research have several limitations. Firstly, comparing the evidence from this study with other studies from the literature is limited by the extensive literature on ERI in several contexts which employed varying instruments and scales over years of ERI research (18, 25, 26). Moreover, the evidence of the economic influence and financial struggles on ERI among university students was only captured in the qualitative study and no quantitative data was obtained. It should also be noted that qualitative data was collected in April-May 2018 during Mid-term exams and quantitative data was collected in May-July 2018, which was toward the end of the academic year during final exams. This may have influcences how students perceived their effort and reward. Additionally, this study was not longitudinal, so it did not test and re-test the ERI in university students over a period of time, providing no information on how the ERI model performs under time change and how stable is the instrument. This is important as there were reports in the literature on the time invariance of the ERI model (7). Future work may extend on this study by providing evidence on the model invariance and stability and its association with poor health reported in this study. Moreover, this study recruited students from one university only, and therefore selection bias cannot be ruled out. Hence, the results of this study cannot be generalized to other students at different universities in Jordan.

In conclusion, the ERI model can be used to measure stress among university students, and to generate evidence on ERI association with several alarming health-related issues. There is an urgent need to develop training programs that offer effective strategies for students to help them cope with their academic-related stress. Moreover, any interventional programs can be targeted at reducing academic-related stress by involving all relevant parities, including university officials, granting agencies and families of students. This study demonstrates that any efforts aimed solely at the students, will not yield optimal results.

## Data Availability Statement

The original contributions presented in the study are included in the article/supplementary material, further inquiries can be directed to the corresponding author/s.

## Ethics Statement

The studies involving human participants were reviewed and approved by King Abdallah University Hospital Institutional Review Board at Jordan University of Science and Technology (Approval number: 15/114/2018). The patients/participants provided their written informed consent to participate in this study.

## Author Contributions

MA conceptualized the study and managed data collection and curation and analysis. TE acquired funding and IRB approvals and supervised data collection. MA drafted the manuscript and TE revised the final manuscript. Both authors contributed to the article and approved the submitted version.

## Funding

TE received a grant from the Deanship of Research, Jordan University of Science and Technology (Grant number: 145/2018) to support fieldwork and data collection. The funders had no role in study design, data collection and analysis, decision to publish, or preparation of the manuscript.

## Conflict of Interest

The authors declare that the research was conducted in the absence of any commercial or financial relationships that could be construed as a potential conflict of interest.

## Publisher's Note

All claims expressed in this article are solely those of the authors and do not necessarily represent those of their affiliated organizations, or those of the publisher, the editors and the reviewers. Any product that may be evaluated in this article, or claim that may be made by its manufacturer, is not guaranteed or endorsed by the publisher.
